# Papillary Muscle Free Strain in Patients with Severe Degenerative and Functional Mitral Regurgitation

**DOI:** 10.5935/abc.20170035

**Published:** 2017-04

**Authors:** Alev Kılıcgedik, Gokhan Kahveci, Ahmet Seyfeddin Gurbuz, Can Yucel Karabay, Ahmet Guler, Suleyman Cagan Efe, Soe Moe Aung, Ugur Arslantas, Serdar Demir, Ibrahim Akin Izgi, Cevat Kirma

**Affiliations:** Kartal Koşuyolu Heart & Research Hospital, Department of Cardiology, Turkey

**Keywords:** Mitral Valve Insufficiency / diagnostic, Mitral Valve Insufficiency / physiopathology, Papillary Muscles / physiopathology, Diagnostic Imaging, Echocardiography / methods, Ventricular Function, Ventricular Remodeling

## Abstract

**Fundamento:**

The role of papillary muscle function in severe mitral regurgitation with preserved and reduced left ventricular ejection fraction and the method of choice to evaluate PM have still been the subjects of controversy.

**Objectives:**

To evaluate and compare papillary muscle function in and between patients with severe degenerative and functional mitral regurgitation by using the free strain method.

**Methods:**

64 patients with severe mitral regurgitation - 39 patients with degenerative mitral regurgitation (DMR group) and 25 patients with severe functional mitral regurgitation (FMR group) - and 30 control subjects (control group) were included in the study. Papillary muscle function was evaluated through the free strain method from apical four chamber images of the anterolateral papillary muscle (APM) and from apical three chamber images of the posteromedial papillary muscle (PPM). Global left ventricular longitudinal and circumferential strains were evaluated by applying 2D speckle tracking imaging.

**Results:**

Global left ventricular longitudinal strain (DMR group, -17 [-14.2/-20]; FMR group, -9 [-7/-10.7]; control group, -20 [-18/-21] p < 0.001), global left ventricular circumferential strain (DMR group, -20 [-14.5/-22.7]; FMR group, -10 [-7/-12]; control group, -23 [-21/-27.5] p < 0.001) and papillary musle strains (PPMS; DMR group, -30.5 [-24/-46.7]; FMR group, -18 [-12/-30]; control group; -43 [-34.5/-39.5] p < 0.001; APMS; DMR group, (-35 [-23.5/-43]; FMR group, -20 [-13.5/-26]; control group, -40 [-32.5/-48] p < 0.001) were significantly different among all groups. APMS and PPMS were highly correlated with LVEF (p < 0.001, p < 0.001; respectively), GLS (p < 0.001, p < 0.001; respectively) and GCS (p < 0.001, p < 0.00; respectively) of LV among all groups. No correlation was found between papillary muscle strains and effective orifice area (EOA) in both groups of severe mitral regurgitation.

**Conclusions:**

Measuring papillary muscle longitudinal strain by the free strain method is practical and applicable. Papillary muscle dysfunction plays a small role in severe MR due to degenerative or functional causes and papillary muscle functions in general seems to follow left ventricular function. PPM is the most affected PM in severe mitral regurgitation in both groups of DMR and FMR.

## Introduction

Mitral regurgitation (MR) is one of the most common valve diseases in developed countries. The main etiologies of MR are classified as degenerative, dilatative and ischemic.^1^ Severe MR may compromise left ventricular function and worsen patients' prognosis.^2^ The mitral subvalvular apparatus contributes significantly to left ventricular function and occurrence of mitral regurgitation. The impairment of the subvalvular apparatus is detrimental to the left ventricular systolic function and mitral regurgitation.^3,4^ Papillary muscle dysfunction has previously been shown as a mechanical cause of mitral regurgitation in patients with functional mitral regurgitation (FMR) in some studies, but, in some others, no correlation was found between mitral regurgitation and papillary muscle dysfunction and even an attenuating effect of papillary muscle dysfunction was reported in most studies.^2,3,5-8^ In experimental studies, under the range of normal loading and inotropic conditions, papillary muscle contraction normally follows the general characteristics of left ventricular contraction,^4^ but ischemia or stunning may disrupt this course. It is reported that in ischemic mitral regurgitation, diminished papillary muscle shortening, which is termed as papillary muscle dysfunction, paradoxically decreases the degree of MR.^5,7^ In patients with normal LV function and MR, fractional shortening has been shown to be normal, similarly to patients with mild or more severe MR.^3^ In patients with degenerative mitral regurgitation (DMR), there is no sufficient knowledge about the role of papillary muscle (PM) dysfunction. The role of papillary muscle function in severe MR and the method of choice to evaluate PM are still controversial.^3,7^ The objective of this study is to evaluate and compare papillary muscle functions in and between patients with severe DMR and severe FMR by using the free strain method.

## Methods

### Study Population

This is a prospective study and included 64 patients with severe MR who were referred for echocardiographic examination at the Kartal Kosuyolu Heart Education and Research Hospital between January 2014 and April 2015. A total of 39 patients had degenerative severe MR (DMR group) and 25 patients had functional severe MR (FMR group). The control group consisted of 30 subjects with no MR and normal ejection fraction. Patients with DMR (mitral valve prolapse, chordae tendinea rupture) and normal ejection fraction (> 60%), and patients with ischemic or non-ischemic FMR with ejection fraction < 40% were enrolled in the study prospectively. In the DMR group, 6 patients had anterior leaflet prolapsus, 26 patients had posterior prolapsus (18 patients with P2 scallop prolapsus, 4 patients with P1 scallop prolapsus and 4 patients with P3 scallop prolapsus) and 6 patients with Barlow's disease. In the FMR group, 21 patients had ischemic heart disease that did not require revascularization, and 4 patients had non-ischemic dilated heart disease. Patients with organic MR caused by other reasons, including rheumatic or senile degenerative heart valve disease, mitral annular calcification, infective endocarditis, and patients with reduced ejection fraction were excluded from the study. Only patients with appropriate echocardiographic images were included in the study. The Local Ethics Committee approved this study.

### Standard Echocardiography

Standard echocardiographic evaluations were performed using a 1 to 5 MHz X5-1 transducer (iE33, Philips Healthcare Inc., Andover, MA). Patients were examined in the left lateral position. Measurements were averaged over 3 consecutive heart cycles. All standard 2D transthoracic echocardographic images from parasternal long axis, short axis, apical four, three and two chamber views, color Doppler and Tissue Doppler images were stored in cine loop format triggered to the QRS complex. Left ventricular diastolic and systolic diameters were measured using M-mode or 2-dimensional echocardiography. Left ventricular ejection fraction (LVEF) was calculated according to Simpson's formula employing a two-dimensional image of the LV chamber during systole and diastole in the four- and two-chamber apical views.

The quantification of MR was assessed as recommended.^9^ The proximal isovelocity surface area (PISA) was visualized from apical four-chamber view. The radius of the PISA was measured at mid-systole using the first aliasing. Regurgitant volume (RV) and effective orifice area (EOA) were obtained using the standard formula. For DMR; RV > 60 mL/beat or EOA > 0.4 cm^2^, and for FMR; RV > 30 mL and EOA > 0.2 cm^2^ were considered as severe MR.^10^ The configuration of mitral leaflets was assessed from the parasternal long axis and apical views. In addition to 2D transthoracic echocardiographic views, all patients with severe DMR underwent 2D and 3D transesophageal echocardiographic (TEE), which provided precise information on type and extent of anatomical lesions, mechanism of regurgitation, etiology and reparability of the valve. The mitral annular diameter seen from the bi-commissural view (MAbic) was measured by conventional 2D TEE at 60-75 degrees and anterior-posterior diameter (MAap) was measured at 120 degrees in the parasternal long-axis view. Anterior and posterior leaflet lengths were measured in diastole at 120º.

Speckle Tracking Echocardiography (STE): Left ventricular strain (circumferential and longitudinal) was evaluated using 2D speckle-tracking imaging. Global circumferential strain (GCS) was assessed from parasternal short axis views of the left ventricle at three levels (base-mid-apical). Global longitudinal strain (GLS) was assessed from apical four, three and two chamber views.

Longitudinal myocardial strain of PMs was evaluated using the free strain method from apical four chamber view for anterolateral PM (APM) and apical long axis view for posteromedial PM (PPM). Patients in whom PM views were visually clear in both systole and diastole were considered eligible for the assessment. Of 110 patients, 15% were excluded from the study because of inadequate image quality.

Free strain is an application of the commercially available software program of Philips (CMQ Q-app). This method enables the measurement of user defined custom local velocities, displacement and deformation using unlimited directional chords display technic. This workflow measures strain within the myocardial region, free of restraints on the location or direction of the measurements, which can be radial, longitudinal, and circumferential. Free strain is thought to be an easy, quick and practical method of measuring myocardial deformation. This method may be particularly preferable in measuring the deformation of PMs since these structures are relatively separate from the LV myocardium and are not included in the commercially available LV strain models.

In order to measure the longitudinal strain by using the free strain method, a region of interest should be selected by clicking two points manually. The first point was selected from the base of the PM at its attachment zone to the LV wall. The second point was selected from the tip of the PM with special attention to keep a 3-5 mm distance from the chordae in order to avoid artifacts.

All STE acquisitions were performed at frame rates between 50-70 Hz frames per second. The average value of strain was taken from the three consecutive beats. The peak systolic values were recorded for GCS, GLS and longitudinal S of APM and PPM.

**Figure 1:**
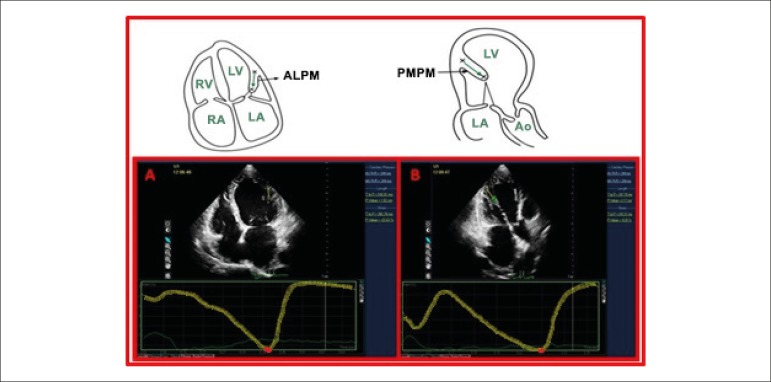
A) Free strain measurement of the APM from apical four chamber image in a patient with FMR. B) Free strain measurement of PPM from apical long axis image of the same patient.

The details of the longitudinal strain measurement with the "free strain" method for both PMs are presented in Figure 1.

### Statistical analysis:

Data management and analysis were performed using IBM SPSS Statistics 16.0 (SPSS, Chicago, IL) software. Continuous variables are expressed as mean (SD) or median (25th to 75th interquartile range [IR]) depending upon variable distribution. Normal distribution was analyzed using the Kolmogorov-Smirnov test. Categorical variables are presented by absolute and percentage numbers and compared using Chi-Square or Fisher's Exact test as appropriate. One-way ANOVA with Tukey post hoc was used to compare continuous variables among groups; when homogeneity of variance was not present, the Kruskal-Wallis test was used for nonparametric independent samples. Mann-Whitney test for nonparametric independent samples for inter-group comparisons were performed to confirm significance. Correlations were tested by Pearson or Spearman's correlation tests, as appropriate.

A p value < 0.05 was considered statistically significant.

## Results

Demographic characteristics of the study population are presented in Table 1. Age and gender were similar in all groups. Standard echocardiographic and STE characteristics are presented in Tables 2 and 3. LA and LV diameters were statistically different among all groups. Atrial fibrillation ratio was statistically different between DMR and FMR groups but this did not seem to significantly affect the results of the study.

**Table 1:** Baseline Clinical Characteristics of Study Population

Variable	DMR (n: 39)	FMR (n: 25)	Control (n: 30)	p value
Age, years	52.5 ± 15	57 ± 15	52.7 ± 9.4	0.40
Gender, male	29 (%74)	20 (%80)	20 (%67)	0.58
NYHA class 3-4	10 (%26)	6 (%24)	0 (% 0)	0.011
Creatinine, mg/dL	0.88 ± 0.26	1.2 ± 0.78	0.81 ± 0.15	0.06
DM	4 (%10)	5 (%20)	3 (%10)	0.45
SBP (mmHg)	128.8 ± 6.8	113.4 ± 8	127.6 ± 9.1	< 0.001
DBP (mmHg)	78.2 ± 5.3	71±5.2	80.8 ± 6.1	< 0.001
Chronic AF	2 (5.1%)	11 (44%)	0	< 0.001

DM: Diabetes Mellitus; SBP: systolic blood pressure; DBP: diastolic blood pressure; AF: atrial fibrillation.

**Table 2:** Baseline characteristics of mean and median values of echocardiographic parameters

Groups	DMR Group (n: 39)	FMR Group (n: 25)	Control Group (n: 30)	p value (all groups)	Pα	Pβ	Pγ
LA (cm)	4.18 ± 0.73	4.72 ± 0.79	3.31 ± 0.37	< 0.001	0.008	< 0.001	< 0.001
LVESD (cm)	3.56 ± 0.67	5.14 ± 0.73	2.89 ± 0.40	< 0.001	0.01	< 0.001	< 0.001
LVEDD (cm)	5.80 ± 0.74	6.51 ± 0.81	4.71 ± 0.41	< 0.001	< 0.001	< 0.001	< 0.001
LVEF (%)	64.5 ± 2.02	33.4 ± 9.06	65.1 ± 1.94	< 0.001	< 0.001	0.22	< 0.001
EOA (cm^2^)	68.75 ± 27.23	33.43 ± 11.03		< 0.001			
RV (ml)	95.97 ± 30.6	50.3 ± 13.9		< 0.001			
AL LENGHT (mm)	27.8 ± 6.41	25.8 ± 3.6		0.465			
PL LENGHT (mm)	17.5 ± 4.15	14 ± 1.41		0.049			
MAbic (mm)	46.6 ± 7.13	33.5 ± 14.8		0.001			
MAap (mm)	41.0 ± 5.62	34.6 ± 1.86		0.009			

LA: left atrium; LVESD: left ventricular end systolic diameter; LVEDD: left ventricular end diastolic diameter; LVEF: left ventricular ejection fraction; PISA: proximal velosity surface area; RV: regurgitant volume; AL LENGHT: anterior leaflet length; PL LENGHT: posterior leaflet length; MAbic: bi-commissural mitral annulus diameter; MAap: A2P2 mitral annulus diameter; Pα: p value of comparing groups DMR-FMR; Pβ:p value of comparing groups DMR-control; Pγ: p value of comparing groups FMR-control.

**Table 3:** Strain values of study population

Groups	DMR (n: 39)	FMR (n: 25)	Control (n: 30)	P value (all groups)	Pα	Pβ	Pγ
GCS (%)	-20 (-14.5/ -22.7)	-10 (-7/-12)	-23 (-21/-27.5)	<0.001	<0.001	0.002	<0.001
GLST (%)	-17 (-14.2/-20)	-9 (-7/-10.7)	-20 (-18/-21)	<0.001	<0.001	0.005	<0.001
APMS (%)	-35 (-23.5/-43)	-20 (-13.5/-26)	-40 (-32.5/-48)	<0.001	<0.001	0.102	<0.001
PPMS (%)	-30.5 (-24/-46.7)	-18 (-12/-30)	-43 (-34.5/-39.5)	<0.001	<0.001	0.012	<0.001

GCS: global circumferential left ventricular strain; GLS: global longitudinal left ventricular strain; APMS: anterolateral papillary muscle strain from apical four chamber; PPMS: posteromedial papillary muscle strain from apical long axis; Pα: p value of comparing groups DMR-FMR; Pβ: p value of comparing groups DMR-control; Pγ: p value of comparing groups FMR-control.

**Figure 2:**
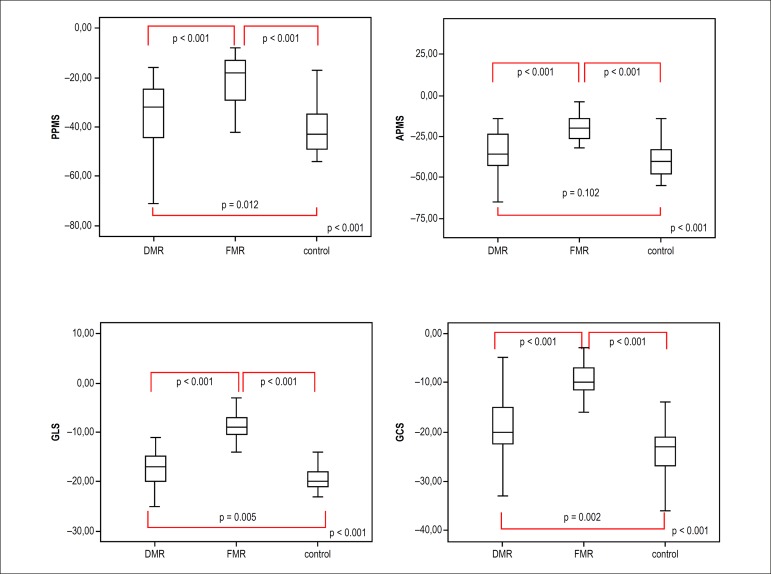
Box-plot values of median (IQR) for APMS, PPMS, GCS, GLS values according to the groups DMR, FMR and controls.

Global left ventricular longitudinal strain and PM longitudinal strains were significantly different among all groups. Posteromedial PM strain (PPMS) of the control group was better than PPMS of the DMR and FMR groups. There was no significant difference in anterolateral PM strain (APMS) between the DMR and control groups, and both strains of the FMR group were significantly lower than PM longitudinal strains of the DMR and control groups. PPMS had the lowest values in both MR groups. Global left ventricular longitudinal and circumferential strains of all three groups followed the same order as PPMS, and were better in the control group than in the DMR group, and the DMR group was better than the FMR group (Figure 2).

APMS and PPMS were highly correlated to LVEF (both p < 0.001), GLS (both p < 0.001) and GCS (both p < 0.001) of the LV among all groups.

No correlation was found between PM strains and EOA in either group with severe MR.

In the DMR group, there was no statistical correlation between PM longitudinal strains and EOA. Any scallop prolapse in the anterior leaflet versus posterior leaflet was correlated to APMS (p = 0.04). Moreover, there was a moderate correlation between the left ventricular end diastolic diameter (LVEDD) and EOA (r = 0.38, p = 0.02). APMS and PPMS were not correlated with LVEF (p = 0.55, p = 0.13; respectively), GLS (p = 0.62, p = 0.54; respectively) and GCS (p = 0.77, p = 0.38; respectively).

In the FMR group, there was also no correlation between EOA and PM longitudinal strains. MAbic was negatively correlated with APMS (r = -0.76, p = 0.03). Posterior leaflet length was correlated with PPMS (r = 0.88, p = 0.01). APMS and PPMS were not correlated with LVEF (p = 0.18, p = 0.09; respectively), GLS (p = 0.33, p = 0.33; respectively) and GCS (p = 0.83, p = 0.93; respectively).

Also, the in the control group, APMS and PPMS were not correlated to LVEF (p = 0.80, p = 0.65; respectively), GLS (p = 0.25, p = 0.43; respectively) and GCS (p = 0.63, p = 0.85; respectively).

## Discussion

Our study demonstrated that PM functions acts in a manner similar to the left ventricle, and is diminished in severe degenerative and functional MR similarly to global ventricular strain. PPMS of the control group was better than in the DMR group and the PPMS of the DMR group was better than in the FMR group. APMS of the control group was similar to the DMR group and better than the FMR group. Although patients had normal ejection fraction in the DMR group, PM longitudinal strain values ordinarily followed GLS and GCS, which were diminished when compared to control group, reflecting a latent systolic dysfunction in the DMR group. Also, in the FMR group, PM longitudinal strain values were well-matched to the diminished global longitudinal and circumferential strain of the impaired LV. No correlation was found between PM free strains and EOA in either group with severe MR. Kisanuki et al. showed that the occurrence of moderate to severe MR was significantly more frequent in patients with combined anterior and posterior PM dysfunction than in those with isolated PM dysfunction or normal PM function. However, they supposed that isolated or combined PM dysfunction was not the only cause for MR unless it was together with left ventricular wall motion abnormalities.^11^ It has been shown in experimental studies that selective paresis of the PMs does not affect the competence of the mitral valve and does not cause MR in a normally contracting ventricle.^11,12^ The main mechanism of MR in FMR is increased tethering forces and reduced coaptation of the mitral valve leaflets by medial/ lateral and apical displacement of the PMs.^13,14^ Tigen et al. ^6^ demonstrated that PM desynchrony was the independent predictor of moderate or moderate-to-severe MR in patients with non-ischemic dilated cardiomyopathy. But in another study that included patients with ischemic and non-ischemic cardiomyopathy, the circumferential strain of PM was evaluated and a straight relationship between PM desynchrony and MR degree was not found; conversely, an inverse relationship between PM longitudinal strain and the degree of MR was found in patients with basal inferior LV remodeling.^2^ There are several studies supporting the paradoxical decrease in ischemic MR by PM dysfunction.^5,7^ This is attributed to a decreased shortening of PMs resulting in the reduction of tethering and MR by PM dysfunction. Although some studies show acute improvement of MR with cardiac resynchronization therapy, the main mechanism is ambiguous and it is considered that if there is PM desynchrony with left ventricular desynchrony, improved coordination of PM contraction can cause acute improvement of MR.^15,16^ Also, in patients with normal ejection fraction, it is reported that PM dysfunction had no significant role in the occurrence of MR.^3^

Our study demonstrated that in the DMR group, APMS was similar to the control group and better than PPMS. In degenerative mitral valve disease, perivalvular ventricular fibrosis and PM fibrosis have been shown in some pathological and MRI studies.^17^ Foci of necrosis are also common in patients with recent onset severe valvular regurgitation, and, in our study, most of the patients had chordal rupture with prolapse. Necrosis or fibrosis may be either focal or diffuse and can involve only one PM or both. The APM is slightly larger and has a richer blood supply than the PPM. Thus, if only one PM contains foci of fibrosis, it is almost always the PPM.^18^ Moreover, combined PM dysfunction is frequently seen in patients with FMR, in contrast to patients with apparent mitral valve prolapse in which combined PM dysfunction was noted in a small number of patients.^8^

In addition, in our study, any scallop prolapse on anterior leaflet was associated to a decreased APMS value when compared to posterior leaflet prolapse. This may be because the anterior leaflet is larger, longer and usually thicker than the posterior leaflet. The posterior leaflet is crescent-shaped with a short radial length and a long circumferential base.^19,20^ Thus, severe MR may cause less shortening of PM in systole caused by redundant anterior leaflet movement towards the left atrium by the driving force of the mitral regurgitant jet. In addition, the mitral annulus is a nonplanar saddle-shaped structure. The anterior portion of the mitral annulus is continuous with the rigid aortic annulus and is elevated towards the atrium as a 'horn'. However, the posterior mitral annulus is more flexible, allowing a systolic apical bending along a commissural axis. This helps to reduce tissue stress.^19,21^ Anterior leaflet prolapse may be more associated with increased tissue stress than with posterior prolapse.

In the FMR group, an increase in MAbic is associated with decreased APMS. When the LV dilates, the mitral annulus also dilates and flattens, loses its saddle shape and systolic annular contraction. This causes malcoaptation of mitral leaflets,^22,23^ and an increase in tethering forces resulting in less shortening of PMs. In addition, we found that posterior leaflet length was associated with PPMS. In FMR, tethering of the mitral leaflets is often on the posterior leaflet and particularly on the posteromedial scallop.^24^ According to this finding, as the posterior leaflet length increases, tethering of the mitral leaflet diminishes and PM function improves.

As far as we know, ours is the only study that compares PM function in degenerative and functional severe MR patients by using the free strain method. In previous studies, longitudinal and circumferential strain methods were used to evaluate PM function. We used the free strain method to measure the longitudinal strain of two points on the PMs, which seems easier and more practical in clinical use, although there is no standard guideline on free strain in PM function evaluation so far.

Some studies with animals have shown that under the range of normal loading and inotropic conditions, PM dynamics closely follow the dynamics of the LV as a whole. They shorten during ejection like the rest of LV, and their lengths change only very slightly during the isovolumic periods. During isovolumic contraction they shorten slightly and during isovolumic relaxation they lenghten slightly. Under ischemic conditions, the dynamic behaviour of PMs reverse during isovolumic contraction and isovolumic relaxation.^4,25,26^ In the study by Kisanuki et al., fractional shortening of PMs was calculated by using end diastolic and end systolic length of PMs on 2D TTE.^11^ In our study, the values of longitudinal strain of PMs, using free strain method, were correlated with their values of fractional shortening of PMs.

### Limitations

Only patients with severe MR were included in the study. Patients with mild or moderate MR were excluded. We evaluated PM function in severe MR comparing and associating with EOA in DMR and FMR patients. The behavior of free strain patterns in patients with mild or moderate MR is unknown. We used the free strain method to evaluate PM function, but there is no standard usage or values about this method. Since this is study with a small population, the present results should be confirmed in further studies with a larger number of patients.

## Conclusion

Our study, in accordance with previous studies, has demonstrated that PM dysfunction plays a small role in severe MR due to degenerative or functional causes, and PM function in general seems to follow LV function. PPM is the most affected PM and has the lowest longitudinal strain values in both severe MR groups. Free strain is a practical and applicable method of choice to measure PM longitudinal strain.
